# Case Report: Acute superior mesenteric vein and portal vein thrombosis complicated by small intestinal necrosis: a case of successful endovascular treatment

**DOI:** 10.3389/fmed.2026.1840626

**Published:** 2026-07-14

**Authors:** Hualin Li, Zihan Liu, Rongchao Xiang, Xin Li

**Affiliations:** 1 Center for Gastroesophageal Disease, Deyang People’s Hospital, Deyang, Sichuan, China; 2 Center for Vascular Intervention, Deyang People’s Hospital, Deyang, Sichuan, China

**Keywords:** acute portal vein thrombosis, acute superior mesenteric venous thrombosis, case report, endovascular therapy, intestinal congestion and necrosis

## Abstract

**Background:**

Acute superior mesenteric vein thrombosis (ASMVT) and acute portal vein thrombosis (APVT) are rare conditions. In the absence of timely diagnosis and treatment, they can lead to intestinal congestion and necrosis, sepsis, and death. When intestinal necrosis is clinically suspected, emergency exploratory laparotomy is the standard of care and may necessitate resection of the necrotic intestine. Standard operating procedure (SOP) and guidelines remain lacking due to the rarity of this disease.

**Case Description:**

This article reports the case of a 44-year-old male patient who presented with acute abdominal pain and was diagnosed with acute complete thrombosis of the superior mesenteric vein and portal vein, with a clinical diagnosis of small intestinal congestion and necrosis. Emergency endovascular intervention was performed: percutaneous transhepatic portal venography, balloon dilation, thrombolysis and thrombectomy, and indwelling catheter thrombolysis. Postoperatively, the patient was transferred to the intensive care unit (ICU) for continued with catheter-directed thrombolysis (CDT) and anticoagulation therapy. Vascular recanalization occurred on postoperative day 9, and the patient was discharged after an uneventful recovery, thereby obviating the need for surgical intervention and bowel resection.

**Conclusion:**

For acute superior mesenteric vein and portal vein thrombosis complicated by intestinal congestion and necrosis, endovascular therapy combined with anticoagulation may be a treatment option prior to surgical intervention and warrants further investigation.

## Introduction

ASMVT accounts for 6% to 9% of mesenteric ischemia cases and 1 in 5000 to 15000 hospital admissions (1–3). APVT occurs in approximately 2.7 per 100,000 individuals (1, 4, 5). Acute vascular obstruction involving both SMV and PV is associated with a considerable mortality rate in clinical practice. In the context of acute non-cirrhotic, the causes of ASMVT and APVT include cirrhosis, abdominal infection or inflammation, surgical procedures, deficiencies in antithrombin III, protein C, protein S, and factor V Leiden, as well as malignancy (6–10).

For patients presenting with hematochezia, signs of peritonitis, and contrast-enhanced CT findings indicative of intestinal necrosis, emergency exploratory laparotomy combined with systemic anticoagulation represents the traditional approach (3, 11–14). However, intraoperatively determining whether the bowel is truly necrotic and establishing the extent of intestinal necrosis remain challenging. To ensure safety, extensive bowel resection with enterostomy may be performed, and multiple surgical procedures may be required within a short period. This significantly impairs the patient’s quality of life, with the surgical mortality rate for SMVT reported as 29%–38% (2).

Endovascular techniques, particularly CDT, offer a less invasive alternative that may achieve early recanalization and avoid extensive bowel resection. Given the rarity of these conditions, high-level evidence remains limited. We report a case of ASMVT and APVT managed with an endovascular-first strategy, aiming to contribute to the evidence base for this evolving treatment paradigm.

## Case presentation

### Patient information

A 44-year-old male presented to the emergency department with a 1-day history of upper abdominal distension and pain, accompanied by hematochezia and vomiting. One day prior to admission, the patient experienced persistent, intolerable upper abdominal distension and pain without an identifiable cause. During treatment at a local hospital, he experienced five episodes of vomiting gastric contents without hematemesis, followed by four episodes of hematochezia (approximately 250–300mL of dark red blood each) and self-reported decreased flatus. He was subsequently transferred to our emergency department. The patient endorsed a history of mild depression and denied any personal or family history of coagulation or bleeding disorders, and the review of systems was otherwise unremarkable. Physical examination revealed abdominal distension, increased abdominal rigidity, diffuse tenderness, rebound tenderness, and guarding, along with markedly diminished bowel sounds.

### Diagnostic assessment

Contrast-enhanced abdominal CT revealed complete thrombosis of the main portal vein, the right branch, and the superior mesenteric vein and its tributaries, with suspected congestive necrosis in the mid-small intestine (Figures 1A1–D1). Vital signs and laboratory values upon admission are presented in Table 1.

**FIGURE 1:**
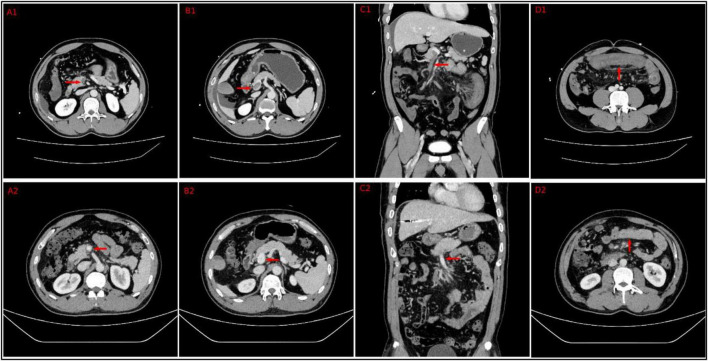
Contrast-enhanced CT images before and after endovascular surgery. **A1–D1** are preoperative images, and **A2–D2** are postoperative images. **(A1)** Complete filling defect in the main trunk of the superior mesenteric vein, suggestive of thrombosis. **(B1)** Complete filling defect in the main portal vein, suggestive of thrombosis, with ascites. **(C1)** Coronal image shows complete filling defect in the main trunk of the superior mesenteric vein and poor opacification of its distal branches, suggestive of thrombosis. Perihepatic fluid and markedly reduced enhancement of the small bowel wall are also observed. **(D1)** Venous phase contrast-enhanced CT shows edematous thickening of the small bowel in the mid-abdomen with markedly decreased wall enhancement, suggestive of congestive necrosis. **(A2)** The main trunk of the superior mesenteric vein is well opacified, indicating resolution of thrombosis. **(B2)** The main portal vein is well opacified, indicating resolution of thrombosis. **(C2)** Coronal image shows opacification of the main superior mesenteric vein and its branches, with resolution of thrombosis. Perihepatic ascites is absent, and small bowel wall enhancement is preserved. **(D2)** Venous phase contrast-enhanced CT demonstrates full recovery of perfusion to the small bowel wall in the mid-abdomen.

**TABLE 1:** Vital signs and laboratory values.

Test	Value	Reference range	Unit
Blood pressure	136/75	120−139/ 80−89	mmHg
Heart rate	76	60−100	Beats/minute
Oxygen saturation	98	> 95	Percent at room air
Temperature	36.50	36.10−37.20 (oral)	°Celsius
White blood count	17.07	3.50−9.50	x10^9^/L
Absolute neutrophil count	15.06	1.80−6.30	x10^9^/L
Neutrophil percentage	88.20	40.00−75.00	Percent
High-sensitivity C-reactive protein	40.67	0.50−10.00	mg/L
Procalcitonin	0.266	< 0.076	ng/ml
Hemoglobin	179	130−175	g/L
Total bilirubin	34.10	≤ 21.00	umol/L
Dotal bilirubin	7.20	≤ 6.80	umol/L
Aspartate aminotransferase	33	15−40	U/L
Alanine aminotransferase	18	9−50	U/L
Alkaline phosphatase	85	45−125	U/L
Blood glucose	8.35	3.90−6.10	mmol/L
Creatinine	101.10	57.00–97.00	umol/L
Lactate	3.00	0.50−1.60	mmol/L
Lactate dehydrogenase	244	109–245	U/L
Uric acid	451	208−428	umol/L
Glomerular filtration rate	81.02	80.00−120.00	ml/min
Prothrombin time	15.10	10.00−14.00	second
Activated partial thromboplastin time	32.30	23.00–35.00	second
International Normalized Ratio	1.29	0.80−1.29	
Fibrinogen	3.90	2.00−4.00	g/L
Fibrinogen degradation products	59.92	0.00−5.00	mg/L
D-Dimer	18.99	0.00−0.50	mg/L

### Therapeutic intervention

Upon admission, the patient received cefoperazone-sulbactam as antibiotic therapy and was administered intravenous fluids. Given the presence of active hematochezia on admission, systemic anticoagulation was briefly deferred until the endovascular procedure could be performed.. At 2 h 5 min after admission, the patient underwent percutaneous transhepatic portal venography, balloon dilation, thrombolysis and thrombectomy and CDT of the superior mesenteric vein (Figures 2A, B). During the procedure, a loading dose of urokinase (200,000 U) was injected as a bolus via the indwelling catheter in the superior mesenteric vein. Postoperatively, the patient was transferred to the ICU for monitoring and management. Continuous CDT was maintained with urokinase at a dose of 400,000 U per 24 h (diluted in normal saline and delivered as a continuous intravenous infusion) for a total of 5 days. Throughout the thrombolysis period, coagulation parameters were strictly monitored: fibrinogen and D-dimer were measured every 6–8 h and activated partial thromboplastin time (APTT) was measured every 12 h, with the infusion rate adjusted dynamically to maintain the fibrinogen level above 1.0 g/L. No bleeding complications, such as puncture site oozing, hematuria, or mucosal bleeding, were observed. Subcutaneous enoxaparin (6,000 AXaIU q12 h) was administered for anticoagulation, cefoperazone-sulbactam for infection, and total parenteral nutrition (TPN). On postoperative day 1, the patient’s symptoms of abdominal pain and hematochezia significantly improved. Abdominal rigidity gradually decreased, and tenderness and rebound tenderness also showed marked improvement compared with previous findings. On postoperative day 3, the patient was transferred from the ICU to the general ward, where the aforementioned treatment regimen was continued. Catheter-directed venography via the superior mesenteric vein was performed on postoperative days 3 and 6 (Figures 2C, D). Follow-up contrast-enhanced abdominal CT was performed on postoperative day 7 (Figure 1A2–D2). Both demonstrating progressive reduction of thrombus and gradual restoration of patency in the portal vein and superior mesenteric vein. On postoperative day 7, the patient began oral intake and was discharged on day 9. After discharge, oral rivaroxaban (15 mg, bid) was continued for anticoagulation.

**FIGURE 2:**
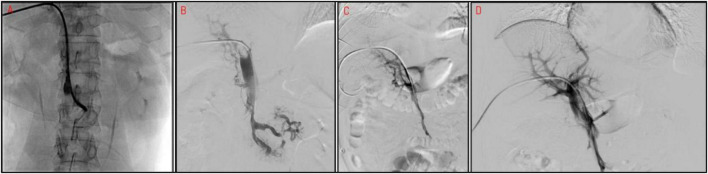
Digital subtraction angiography (DSA) images before and after endovascular treatment. **(A)** Intraoperative percutaneous transhepatic portography shows filling defects and poor opacification of the superior mesenteric vein, main portal vein, and branches, suggestive of multiple thrombi. **(B)** Following intraoperative thrombectomy and aspiration, the portal vein, superior mesenteric vein, and some branches are opacified, with marked improvement in blood flow compared with baseline. **(C)** Angiography on postoperative day 3 shows opacification of the main superior mesenteric vein and near-complete thrombus resolution. Residual thrombus in the main portal vein and collateral circulation are present. **(D)** Angiography on postoperative day 5 shows good opacification of the superior mesenteric vein and main portal vein, with only minimal residual organized thrombus in the portal vein. Blood flow is markedly improved compared with the previous examination.

### Follow-up and outcomes

Following discharge, the patient was maintained on oral rivaroxaban at an initial dose of 15 mg twice daily for the first 3 weeks, and subsequently transitioned to 20 mg once daily for long-term maintenance. During the initial weeks of follow-up, the patient experienced no recurrence of abdominal pain or hematochezia. To date, the patient has been followed for a total of 6 months. Given the extensive and unprovoked nature of the thrombosis, anticoagulation is planned to continue for a minimum of 12 months, with indefinite therapy considered based on annual reassessment of thrombotic and bleeding risks. The surveillance protocol includes outpatient visits every 3 months, together with Doppler ultrasound of the portal venous system and coagulation studies. At the most recent follow-up, the patient remained asymptomatic, and Doppler ultrasound confirmed sustained recanalization of both the portal vein and the superior mesenteric vein. The patient expressed satisfaction with the clinical outcome and has remained adherent to the anticoagulation regimen while continuing under close surveillance with periodic imaging evaluation. The timeline summary is presented in Table 2.

**TABLE 2:** Timeline summary.

Time	Stage	Event
Days 1−2	Initial presentation	The patient presented with abdominal distension, pain, hematochezia, and vomiting. Examination revealed diffuse tenderness, rebound tenderness, guarding, and markedly diminished bowel sounds. (Admission: February 10, 2026, 22:59)
	Diagnostic workup	Routine blood tests and contrast-enhanced abdominal CT were performed. CT showed complete ASMVT and APVT, along with congestive necrosis of the mid-small intestine.
	Treatment	Emergency endovascular intervention (Surgical duration 01:05—03:15).
Days 2−4	ICU management	CDT of superior mesenteric vein, anticoagulation, anti-infective therapy, and TPN.
Day 5	General ward management	Follow-up portal venography via indwelling catheter; other treatments per ICU protocol.
Day 8	Diagnostic workup	Repeat portal venography via indwelling catheter, embolization of the puncture tract, and discontinuation of thrombolytic therapy.
Day 9	Diagnostic workup	Repeat contrast-enhanced abdominal CT, initiation of oral feeding.
Day 10	Discharge	Continued rivaroxaban anticoagulation post-discharge, regular outpatient follow-up.

## Discussion

Although the incidence of ASMVT and APVT in healthy individuals is low, the associated mortality rate is high, with published reports estimating it at approximately 19% to 23% (15). For ASMVT and APVT complicated by intestinal congestion and necrosis, surgical intervention is the standard treatment. Most surgeons resect bowel segments that are necrotic or deemed highly likely to be necrotic, and most opt for an enterostomy, which significantly impairs the patient’s quality of life. Moreover, intestinal resection does not address the fundamental cause—the thrombus within the superior mesenteric vein and portal vein—and some patients may experience recurrent intestinal necrosis after surgery. Furthermore, standard anticoagulation therapy does not completely resolve thrombotic obstruction, with early recanalization rates reported to be only 35% to 40% (16, 17). Furthermore, the role of thrombolysis in acute portal and mesenteric venous thrombosis (PVST) has also been supported by the literature. Gao et al. reported a patient with acute non-cirrhotic symptomatic extensive PVST who did not respond to anticoagulation and achieved successful portal vein recanalization after a 7-day course of systemic thrombolysis (18). Another systematic review and meta-analysis demonstrated that thrombolytic therapy for PVST achieved an overall response rate of 93%, a complete recanalization rate of 58%, and a 30-day mortality of 4%. However, the 18% bleeding event rate reported in that meta-analysis cannot be overlooked (19). Compared with surgical intervention, anticoagulation and systemic thrombolysis, endovascular treatment achieves faster vascular reconstruction, thereby reducing complications related to intestinal ischemia, necrosis, and portal hypertension (20). Moreover, endovascular treatment allows for CDT and reduces the required systemic dose of thrombolytic agents, thereby potentially reducing bleeding complications (21). A multicenter study by Rössle et al. compared medical therapy with interventional treatment in 65 patients and demonstrated that interventional procedures were associated with a lower procedure-related mortality rate (2.9%) and a higher treatment success rate (54% vs. 17%) (22).

In clinical practice, the decision to prioritize surgical intervention or endovascular therapy should be based on a comprehensive assessment of clinical presentation, laboratory findings, and contrast-enhanced abdominal CT features, along with the patient’s preferences. The present case involves a previously healthy 44-year-old male who initially presented with acute abdominal pain, hematochezia, and peritoneal signs. Contrast-enhanced abdominal CT revealed ASMVT and APVT with suspected small bowel necrosis. Although intestinal necrosis was clinically suspected in this patient, the lactate level was only mildly elevated (3.00 mmol/L), and the lactate dehydrogenase level was at the upper limit of normal (244 U/L). These findings argued against the likelihood of established transmural intestinal necrosis, a condition that usually presents with markedly higher levels of both markers. Although contrast-enhanced abdominal CT demonstrated extensive thrombosis involving the main portal vein, its right branch, and the superior mesenteric vein and its tributaries, the bowel wall changes were described as “suspected” congestive necrosis rather than definitive findings, and no evidence of perforation or pneumatosis intestinalis was identified. These observations suggested that the ischemic bowel injury might remain reversible, provided that prompt vascular recanalization could be achieved. Moreover, because the patient presented with peritoneal signs and hematochezia, emergency surgery remained a possibility at any time. Systemic thrombolysis was considered unsafe, given the prohibitively high risk of hemorrhage from potential bowel resection or mucosal disruption. In addition, compared with CDT, systemic thrombolysis requires a longer time to achieve vascular recanalization, raising concerns that irreversible intestinal necrosis might develop during this prolonged course of treatment. Furthermore, the patient remained hemodynamically stable at that time. We presented both endovascular therapy and emergency exploratory laparotomy as treatment options, and the patient and family opted to proceed with endovascular therapy first, with surgical intervention reserved as a salvage strategy should the endovascular approach prove unsuccessful. It was on the basis of this comprehensive assessment—balancing the severe thrombotic burden against the absence of definitive signs of irreversible bowel necrosis, together with the patient’s preference—that we prioritized endovascular treatment over systemic thrombolysis and emergency exploratory laparotomy.

Acute non-cirrhotic splanchnic vein thrombosis differs fundamentally from that occurring in cirrhosis, which is primarily driven by portal hypertension and altered hepatic hemodynamics (23). Furthermore, the two conditions differ in their treatment priorities, recommended duration of anticoagulation, and prognosis. Although most guidelines do not recommend thrombolytic therapy in patients with cirrhosis and APVT, Primignani et al., after discussing various potential thrombolysis approaches (including local or systemic thrombolysis, thrombolysis alone or combined with mechanical thrombus removal procedures), nevertheless considered that for patients who do not respond to anticoagulation, thrombolysis may serve as a salvage therapeutic option, provided that the appropriate timing is maintained—preferably within 30 days and, if possible, within 14 days from the acute onset of portal vein thrombosis (24). Current literature recommends comprehensive thrombophilia screening in non-cirrhotic patients, as an underlying prothrombotic state is identified in a substantial proportion of cases (25, 26). In this case, the patient had no local risk factors, including cirrhosis, pancreatitis, malignancy, or recent surgery, and no personal or family history of venous thromboembolism. A comprehensive thrombophilia workup was not performed during the acute phase because acute thrombosis and concurrent anticoagulation confound the results of natural anticoagulant assays. Nevertheless, given the extensive and unprovoked nature of the thrombosis, extended anticoagulation—potentially lifelong—is planned, in line with current expert consensus for extensive unprovoked splanchnic vein thrombosis (25).

The diagnosis of ASMVT and APVT was established in this case with suspected intestinal necrosis. Endovascular therapy was chosen through a shared decision-making process between the clinicians and the patient, achieving rapid vascular recanalization and early discharge. A recent study from the Mayo Clinic (27) analyzed 24 patients with SMVT, including 19 patients presenting with acute thrombosis (79.2%) and 5 with acute-on-chronic thrombosis (20.8%). Interventions included thrombectomy (*n* = 12, 50.0%) and CDT (*n* = 10, 41.7%). Technical success was achieved in 18 patients (75%), with primary patency rates of 88.9% at 14 days and 83.3% at 30 days. The 5-year overall survival rate was 82% (range, 58%–100%). A study by Andraska et al. (28) also demonstrated that among patients who underwent endovascular treatment alone, thrombolysis achieved complete or partial recanalization in up to 91.6% of cases. However, Yang et al. (29) reported that among 13 patients who underwent CDT, 4 required small bowel resection due to segmental bowel infarction and secondary strictures. In contrast, the study population in the aforementioned literature did not exhibit clinical manifestations of intestinal necrosis, which differs from the patient in the present report. This study reports an atypical case managed with unconventional treatment.

Based on the present case and a review of the literature, we propose that in patients with ASMVT/APVT presenting with hematochezia, signs of peritonitis, and CT findings suggestive of intestinal congestion and necrosis, these features do not necessarily indicate complete intestinal necrosis. However, this also raises a clinical challenge: the precise indications for exploratory laparotomy will be more difficult to determine in such cases. Furthermore, conducting large-scale randomized controlled trials for the aforementioned clinical scenario seems not to be feasible, given that ASMVT and APVT are rare diseases with diverse pathogenic mechanisms, and such a trial design would also raise ethical concerns by potentially violating established treatment principles.

## Limitations

Several limitations of this study should be acknowledged. First, as a single case report, the generalizability of our findings is inherently limited. Second, a comprehensive thrombophilia workup was not undertaken during the acute phase because acute thrombosis and concurrent anticoagulation confound the assay results, thereby precluding the identification of an underlying prothrombotic state. Third, the duration of follow-up, although extended since the initial submission, remains relatively short to fully assess the long-term patency of the portal vein and the risk of late sequelae. Additionally, creatine kinase was not measured during the initial laboratory evaluation. Despite these limitations, this case illustrates a successful interventional approach that avoided extensive bowel resection and highlights the importance of a structured, multidisciplinary management strategy.

## Conclusion

For rare conditions such as ASMVT and APVT, the standard treatment for clinically suspected intestinal congestion and necrosis is emergency exploratory laparotomy, however, endovascular therapy combined with anticoagulation may represent a therapeutic option worth considering before surgical intervention, although confirmation through large-sample randomized controlled trials is required.

## Data Availability

The original contributions presented in the study are included in this article/supplementary material, further inquiries can be directed to the corresponding author.

## References

[1] Rhee RY Gloviczki P. Mesenteric venous thrombosis. *Surg Clin North Am*. (1997) 77:327–38. 10.1016/s0039-6109(05)70552-1 9146716

[2] Brunaud L Antunes L Collinet-Adler S Marchal F Ayav A Bresler L et al. Acute mesenteric venous thrombosis: case for nonoperative management. *J Vasc Surg*. (2001) 34:673–9. 10.1067/mva.2001.117331 11668323

[3] Harnik IG Brandt LJ. Mesenteric venous thrombosis. *Vasc Med*. (2010) 15:407–18. 10.1177/1358863X10379673 20926500

[4] Jun KW Kim MH Park KM Chun HJ Hong KC Jeon YS et al. Mechanical thrombectomy-assisted thrombolysis for acute symptomatic portal and superior mesenteric venous thrombosis. *Ann Surg Treat Res*. (2014) 86:334–41. 10.4174/astr.2014.86.6.334 24949327 PMC4062453

[5] Oguslu U Uyanik SA Cenkeri HÇ Atli E Yilmaz B Gümüş B. Transhepatic pharmacomechanical thrombectomy of symptomatic acute noncirrhotic, nonmalignant portomesenteric venous thrombosis: midterm results. *AJR Am J Roentgenol.* (2021) 217:418–25. 10.2214/AJR.20.23150 34036807

[6] Yang Z Zhao Y Chen H Zhang H Tan M Li X et al. Portal vein thrombosis in liver cirrhosis: a review of risk factors and predictive indicators. *J Clin Transl Hepatol*. (2025) 13:857–68. 10.14218/JCTH.2025.00124 41089710 PMC12515608

[7] Fisher NC Wilde JT Roper J Elias E. Deficiency of natural anticoagulant proteins C, S, and antithrombin in portal vein thrombosis: a secondary phenomenon? *Gut*. (2000) 46:534–9. 10.1136/gut.46.4.534 10716684 PMC1727871

[8] Makoto I Usui M Wada H Matsumoto T Ohishi K Shindo A et al. Congenital thrombophilia in patients with superior mesenteric venous thrombosis or portal vein thrombosis. *Clin Appl Thromb Hemost*. (2018) 24:1117–21. 10.1177/1076029618774146 29747524 PMC6714751

[9] Stancu B Chira A Coman HF Mihaileanu FV Ciocan R Gherman CD et al. Intestinal obstruction as initial presentation of idiopathic portal and mesenteric venous thrombosis: diagnosis, management, and literature review. *Diagnostics.* (2024) 14:304. 10.3390/diagnostics14030304 38337820 PMC10855345

[10] Jung HJ Lee SS. Combination of surgical thrombectomy and direct. *Vasc Specialist Int.* (2014) 30:155–8. 10.5758/vsi.2014.30.4.155 26217636 PMC4480313

[11] Singal AK Kamath PS Tefferi A. Mesenteric venous thrombosis. *Mayo Clin Proc*. (2013) 88:285–94. 10.1016/j.mayocp.2013.01.012 23489453

[12] Riva N Donadini MP Dentali F Squizzato A Ageno W. Clinical approach to splanchnic vein thrombosis: risk factors and treatment. *Thromb Res*. (2012) 130:S1–3. 10.1016/j.thromres.2012.08.259 23026649

[13] Bergqvist D Svensson PJ. Treatment of mesenteric vein thrombosis. *Semin Vasc Surg*. (2010) 23:65–8. 10.1053/j.semvascsurg.2009.12.008 20298951

[14] Kumar S Sarr MG Kamath PS. Mesenteric venous thrombosis. *N Engl J Med*. (2001) 345:1683–8. 10.1056/NEJMra010076 11759648

[15] Sulger E Dhaliwal HS Goyal A Anand S. Mesenteric venous thrombosis. *StatPearls [Internet].* Treasure Island, FL: StatPearls Publishing (2022). 29083683

[16] Kumari D. Acute mesenteric and portal vein thrombosis: etiology, diagnosis, and interventional management. *Tech Vasc Interv Radiol*. (2025) 28:101058. 10.1016/j.tvir.2025.101058 40784682

[17] Cheng Q Tree K. Systematic review of thrombolysis therapy in the management of non-cirrhosis-related portal vein thrombosis. *J Gastrointest Surg*. (2021) 25:1579–90. 10.1007/s11605-020-04624-4 33452971

[18] Gao FB Wang L Zhang WX Shao XD Guo XZ Qi XS. Successful treatment of acute symptomatic extensive portal venous system thrombosis by 7-day systemic thrombolysis. *World J Gastrointest Surg*. (2022) 14:1082–5. 10.4240/wjgs.v14.i9.1082 36185556 PMC9521475

[19] Gao F Wang L Pan J Yin Y Wang J Xu X et al. Efficacy and safety of thrombolytic therapy for portal venous system thrombosis: a systematic review and meta-analysis. *J Intern Med*. (2023) 293:212–27. 10.1111/joim.13575 36208172

[20] Wei N Mathy RM Chang DH Loos M Merle U Gauss A et al. Endovascular management of acute superior mesenteric vein thrombosis: a retrospective study on thrombolysis outcomes. *CVIR Endovasc*. (2025) 8:41. 10.1186/s42155-025-00558-7 40366487 PMC12078909

[21] Xiang S Wang XA. Review of the effectiveness and safety of catheter-directed thrombolysis for venous thromboembolism. *J Endovasc Ther*. (2026) 33:569–83. 10.1177/15266028241284470 39463058

[22] Rössle M Bettinger D Trebicka J Klinger C Praktiknjo M Sturm L et al. A prospective, multicentre study in acute non-cirrhotic, non-malignant portal vein thrombosis: comparison of medical and interventional treatment. *Aliment Pharmacol Ther*. (2020) 52:329–39. 10.1111/apt.15811 32506456

[23] Intagliata NM Caldwell SH Tripodi A. Diagnosis, development, and treatment of portal vein thrombosis in patients with and without cirrhosis. *Gastroenterology.* (2019) 156:1582e–99e. 10.1053/j.gastro.2019.01.265 30771355

[24] Primignani M Tosetti G Ierardi AM. Approach to different thrombolysis techniques and timing of thrombolysis in the management of portal vein thrombosis in cirrhotic patients. *J Transl Int Med*. (2023) 11:198–202. 10.2478/jtim-2023-0113 37662891 PMC10474884

[25] Easl. Clinical practice guidelines: vascular diseases of the liver. *J Hepatol*. (2016) 64:179–202. 10.1016/j.jhep.2015.07.040 26516032

[26] Plessier A Darwish-Murad S Hernandez-Guerra M Consigny Y Fabris F Trebicka J et al. Acute portal vein thrombosis unrelated to cirrhosis: a prospective multicenter follow-up study. *Hepatology*. (2010) 51:210–8. 10.1002/hep.23259 19821530

[27] Alnahhal K Toskich BB Nussbaum S Li Z Erben Y Hakaim AG et al. Superior mesenteric venous thrombosis: endovascular management and outcomes. *World J Clin Cases*. (2022) 10:217–26. 10.12998/wjcc.v10.i1.217 35071520 PMC8727247

[28] Andraska E Haga L Li X Avgerinos E Singh M Chaer R et al. Retrograde open mesenteric stenting should be considered as the initial approach to acute mesenteric ischemia. *J Vasc Surg*. (2020) 72:1260–8. 10.1016/j.jvs.2020.02.044 32276014

[29] Yang SF Liu BC Ding WW He CS Wu XJ Li JS. Initial transcatheter thrombolysis for acute superior mesenteric venous thrombosis. *World J Gastroenterol*. (2014) 20:5483–92. 10.3748/wjg.v20.i18.5483 24833878 PMC4017063

